# Measuring the burden of infodemics with a research toolkit for connecting information exposure, trust, and health behaviours

**DOI:** 10.1186/s13690-023-01101-7

**Published:** 2023-06-05

**Authors:** Adam G. Dunn, Tina D. Purnat, Atsuyoshi Ishizumi, Tim Nguyen, Sylvie Briand

**Affiliations:** 1 Biomedical Informatics and Digital Health, Faculty of Medicine and Health, the University of Sydney, Sydney, Australia; 2 Department for Epidemic and Pandemic Preparedness and Prevention, Health Emergencies Programme, World Health Organization, Geneva, Switzerland

**Keywords:** Infodemic, Infodemiology, Health behaviours, Misinformation

## Abstract

**Background:**

During a public health emergency, accurate and useful information can be drowned out by questions, concerns, information voids, conflicting information, and misinformation. Very few studies connect information exposure and trust to health behaviours, which limits available evidence to inform when and where to act to mitigate the burden of infodemics, especially in low resource settings. This research describes the features of a toolkit that can support studies linking information exposure to health behaviours at the individual level.

**Methods:**

To meet the needs of the research community, we determined the functional and non-functional requirements of a research toolkit that can be used in studies measuring topic-specific information exposure and health behaviours. Most data-driven infodemiology research is designed to characterise content rather than measure associations between information exposure and health behaviours. Studies also tend to be limited to specific social media platforms, are unable to capture the breadth of individual information exposure that occur online and offline, and cannot measure differences in trust by information source or content. Studies are also designed very differently, limiting synthesis of results.

**Results:**

We demonstrate a way to address these requirements via a web-based study platform that includes an app that participants use to record topic-specific information exposure, a browser plugin for tracking access to relevant webpages, questionnaires that can be delivered at any time during a study, and app-based incentives for participation such as visual analytics to compare trust levels with other participants. Other features of the platform include the ability to tailor studies to local contexts, ease of use for participants, and frictionless sharing of de-identified data for aggregating individual participant data in international meta-analyses.

**Conclusions:**

Our proposed solution will be able to capture detailed data about information exposure and health behaviour data, standardise study design while simultaneously supporting localisation, and make it easy to synthesise individual participant data across studies. Future research will need to evaluate the toolkit in realistic scenarios to understand the usability of the toolkit for both participants and investigators.

**Supplementary Information:**

The online version contains supplementary material available at 10.1186/s13690-023-01101-7.

## Background

An infodemic is an overflow of information across physical and digital environments during a public health emergency, which makes it difficult for people to find information to better protect themselves and their communities [4]. During an infodemic, timely and reliable communications from trustworthy sources can be undermined by a flood of low-quality sources and misinformation, and challenges in discerning between conflicting information. This creates a challenge for public health responses to the emergency by creating confusion, misunderstanding of health information, or mistrust in health authorities. In the twenty years since infodemiology was defined as an area of research [16], these public health challenges have been observed for vaccine-preventable diseases [2, 17, 20], the uptake of e-cigarettes among non-smokers [1, 32] and the introduction of the human papillomavirus vaccine [7, 12, 24].

WHO has defined an infodemic as an overabundance of information, including misinformation and disinformation, which occurs during a health emergency. While low quality health information is always in circulation, the context of health emergencies is of special interest to the WHO, because people search for, process, react to and use health information differently during crises. Harmonised measures of how people encounter and engage with health information would help us understand how the information environment affects health behaviours at population levels, and how that might be different during and outside of a public health emergency. Other differences may be topic-specific—consider the use of face masks, immunization, drinking alcohol, smoking, taking unproven treatments or diagnostics, and others. Other differences may exist in different community contexts—a pandemic or an epidemic, an immunization campaign in a community of focus, health promotion in a vulnerable group, communities where adverse events during an immunization campaign are widely publicized.

Since the beginning of the COVID-19 pandemic, the World Health Organization (WHO) has considered an expanded definition of infodemiology to study not only information that is produced and consumed online, but also that circulating in offline environments and communities. For research to be actionable in how it informs health emergency preparedness and response, it requires harmonised measures and cohesive interventions that can only be achieved through transdisciplinary approaches [4]. Measures of impact and the interventions they inform must consider online and offline sources of information and account for the complex ways in which information exposure and trust relate to non-protective behaviours and poor health outcomes [3, 28].

Social media analysis is a common study design in infodemiology [30], but very few are designed to link measures of individual information exposure to risks of harmful or non-protective behaviours or health outcomes, or observe or measure trust as a modifying factor. Most social media analyses only measure the incidence of relevant information online and can only speculate on impact [10]. This skewed focus in infodemiology research affects our ability to assess the burden of disease associated with information exposure, and limits the potential to use infodemiology research to inform public health actions aimed at reducing the impact of exposure to low-quality or harmful information on health outcomes [9]. A further challenge comes from a shift away from making data available for academic research on Facebook and Twitter, and other popular social media platforms never making user-level detail accessible from inception. This has introduced new challenges to surveillance that samples social media users to estimate information exposure.

Another challenge comes from inconsistency in how information exposure is measured. Most data-driven infodemiology studies are restricted to individual social media platforms [27, 29]. Where information access, exposure, and engagement are measured, they are measured inconsistently across studies due to differences in sampling and inclusion criteria. Beyond social media, other—potentially more important—sources and conduits of information that might influence behaviour are typically not measured, including targeted advertising, consultations with health professionals, and online and offline conversations between friends and family. To the best of our knowledge, no studies have been conducted to determine whether data from individual social media platforms can be used as a proxy for broader information exposure in the context of identifying risk factors for harmful or non-protective behaviours.

Prior to the rapid growth of social media platforms and data-driven infodemiology studies, measures of information exposure came from surveys. Simple questionnaires would ask participants questions about which information sources they access and trust, and media use diaries could be used to collect more detailed information about some of the sources of information people engaged with by time of day [5]. An advantage of these approaches was that they captured information sources people recall, which means they represent more salient information. Disadvantages included intrusiveness in the sense that they required effort from participants, and that they relied on participants remembering what they have seen, heard, or read days or weeks later.

Recognising this as an issue in infodemiology, the WHO developed research priorities related to measuring the burden of infodemics on population health. The first WHO infodemiology conference discussed these issues and developed research priorities related to measuring the infodemic burden [4]. In a follow-up WHO infodemiology conference, a panel of experts developed recommendations on specific actions that needed to be taken to improve the availability and quality of data required to measure infodemic burden [31].

Recognising the need for new tools in the area, our aim here was to describe one possible solution for measuring associations between information exposure and health behaviours, with a focus on making the tools available and easy to use in low-resource settings.

## Methods

To design a solution for measuring associations between information exposure and health behaviours, we considered existing tools for measuring information exposure and reviewed the advantages and disadvantages relative to a set of requirements for the system. The results then include a proposed solution that addresses the requirements considering the advantages and disadvantages of those tools.

Information exposure includes the information that people access through searching, conversations, or encountered in other situations such as advertising. Methods for collecting these data include asking them to keep track of relevant information they see online and offline (active collection); using online tracking of the sites and webpages they visit while searching or browsing online (passive collection); or estimating what social media users might see on certain social media platforms at scale (population-level collection). Each of these approaches have advantages and disadvantages related to the scope of what they can measure, the potential for bias in the sampling of information exposure per person or across a population, and the effort required of the people who participate in the studies.

Active data collection involves asking people to recall or record information they accessed. Traditional media diaries might ask study participants to recall the broad sources of media they saw or heard and record that information in the form of a timetable. There are some tools that have been developed for collecting media use diaries electronically [23], but none that suit the specific purpose of working with topic-specific health information and measure participant trust in the information they access. The use of smartphones to collect self-reported data in repeated measure designs is relatively common across communications research [25], including for data similar in structure and type to sources and frequency of information access.

Other more recent tools include online tracking tools that study participants use on their personal devices to passively track exposure to information [5]. These tools can be deployed on desktop and mobile devices and are less intrusive compared to media use diaries, but informed consent and data privacy are of critical importance. One advantage of passive tracking is the reduced burden on participants, which might make it feasible to undertake very large studies with thousands or tens of thousands of participants. The experience of NYU Cybersecurity for Democracy with Ad Observatory following their analysis of political advertising [13, 14], suggests that social media platforms may be reluctant to support or allow tools for tracking what users see on their pages. A disadvantage of online tracking is that it does not capture offline exposures and conversations and given that people may use multiple devices and browsers, may only capture a biased portion of a participant’s information exposure.

Measures of information exposure (rather than counting what users post) have been used to examine vaccination and politics [8, 11, 19]. Despite the volume of studies using social media data for health applications, there are relatively few examples where social media user data have been individually linked to health outcomes, and key examples include early detection of depressive episodes from Twitter [6] and predicting certain diagnoses from Facebook [15]. While the ability to capture information exposure with minimal intrusion is a clear advantage, estimates of information exposure reconstructed from social media data are a likely to represent a limited view of an individual’s overall information exposure.

To answer key questions about associations between information exposure and harmful or non-protective health behaviours, we propose the development of new tools for tracking information exposure that meets the following set of requirements. The tools need to be able to individually link measures of information exposure to outcomes from surveys of attitudes, behaviours, or data about diagnoses or health outcomes. The tools must meet the highest international standards for data privacy so the tools can be used in research studies in any jurisdiction, the tools need to be easy for participants to use, and clear information about data privacy needs to be communicated to study participants to meet ethical requirements in an environment where trust is important [X]. To make it easier to synthesise results across studies, the toolkit needs to make it easy for investigators to implement standardised protocols so that studies can be deployed quickly and consistently everywhere, including in low resource settings.

## Results

### Components of a platform for studies connecting information exposure to behaviour

**Fig. 1:**
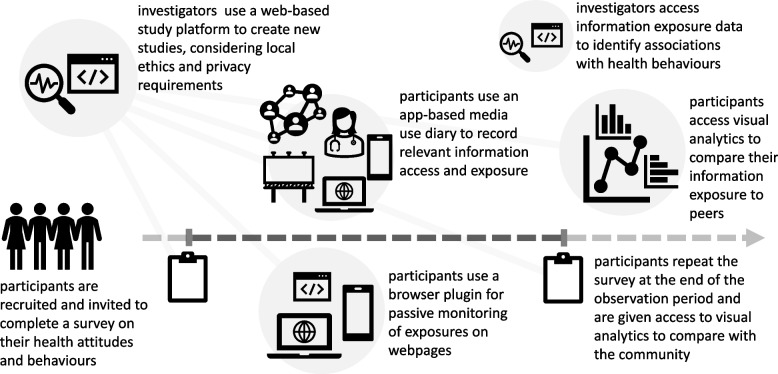
A schematic representation of a proposed toolkit and web-based study platform designed to connect measures of information exposure to health behaviours, standardise study protocols, and support synthesis

One possible solution would include a set of components related to (a) the active and passive tools for collecting data from participants about the topic-relevant information they access; (b) a platform for designing and managing studies that deploy these data collection tools with questionnaires from validated survey instruments that elicit measures of health attitudes, knowledge, or behaviours; and (c) visual analytics that allow participants to compare themselves to the rest of the population at the conclusion of a study as an incentive for participation (Fig. 1). An example of such a solution is described as the Information Diary Platform (IDP) (see Supplementary Information).

### Active data acquisition via an app-based media use diary

The active data collection tool is designed for use in cohort studies that examine the relationship between topic-specific exposure to information and outcomes related to health attitudes or behaviours. It is essentially a modernised version of a media use diary and involves study participants installing a simple app on their smartphone or other devices, using a personalised invitation code to join a study, and then adding details of topic-specific information exposures as they occur during a study period. A typical study might begin with a questionnaire from a validated survey instrument, a study period of weeks to months where study participants record examples of topic-relevant information they see, and a repeat of the questionnaire at the end of the study. Rather than measuring change in attitudes, the expectation is that the information exposures represent a snapshot of a participant’s online and offline information environment, and the questionnaire answers are unlikely to change in that period.

The design of any application for use as a modern media use diary needs to balance the time and effort (for example, the number of clicks and amount of data entry) required of participants with the granularity of the data collected in the study. Minimising time and effort of participants may help avoid participant dropout, while detailed information about the content and user perceptions of information they access or are exposed to during the study period is useful for better characterising risks associated with certain behaviours. For example, we may value detailed information about the content of a social media post promoting and providing information on COVID-19 vaccination, whether they encountered it or searched for it, as well as the level of trust they have in that content because they may be important risk factors associated with health behaviours. One way to implement the collection of more detailed information is to create pre-defined categories related to searching, browsing, and offline interactions, and subcategories describing the specific sources within each category, such as Facebook, consultation with a healthcare provider, or search results on a webpage.

To be useful, the tool must also be simple for study participants to use. One way to support this is to implement a flexible reordering of menu items to make it faster for participants to enter instances of access or exposure. By rearranging the categories and subcategories based on the frequency with which participants report exposures from each of the sources reduces participant effort. For example, if a participant most often engages with topic-specific information on Facebook, then Facebook will move to the top of the subcategory list. This kind of adaptive presentation of options could help to minimise dropout and maximise consistent use. Other considerations include the use of simple and persistent sign-on across multiple devices, which also contributes to ease of use and should help to minimise dropout.

### Passive data acquisition via a browser plugin for tracking online activity

The passive data collection tool is designed to monitor relevant online browsing activity of participants and record information about when they visit webpages with topic-relevant content. The browser plugin monitors the text on the webpages that a user visits on any device with a browser, and records the timing, source, and other metadata of any webpage that includes keywords relevant to the study in which they are enrolled.

Where the active data collection might influence how users engage with relevant information by asking them to actively record what they notice, the passive data collection tool should be able to operate in the background without interfering with the online activity of the user. The advantage of this approach is that the tool is less likely to change the behaviour of the participant as they engage with relevant information, but the disadvantage is that without additional features, it cannot discern levels of engagement and trust with the information.

As an alternative to the passive data acquisition, a hybrid version of the tool can be used to prompt users to answer questions about the topic-relevant information presented on the webpage they are viewing. For example, a pop-up from the browser plugin could ask the user about their level of trust in the topic-relevant information on the webpage they are viewing. While this has the disadvantage of interfering in the online browsing of study participants, it could be used judiciously (for some participants some of the time) to estimate population-level trust in webpages that are visited frequently. Given its potential to influence behaviour, it could also be used only for certain cohorts to evaluate the impact of active data collection relative to passive data collection.

### Study participation incentives via visual comparative analytics

The toolkit uses visual analytics as a non-financial incentive for participating in studies. Financial incentives may not be as useful for encouraging users to keep track of their information exposure completely and in ways that accurately represent their true information exposure. Other types of incentives encourage users to provide complete and accurate data by providing feedback that depends on those data [18, 26]. These kinds of incentives were recommended by researchers with experience in connecting complex social media data to personality outcomes [21]. Avoiding financial incentives might also facilitate deployment of studies in low resource settings.

As an example, visual analytics could present users with a visual summary of their information exposure, including comparisons with other users of the tools during the same period. This could include information about the trustworthiness of the information sources they accessed or exposed to, as well as differences between what they trusted and what other users trusted. Other options might include measures of the frequency of information access and exposure compared to other users by source.

To avoid risks to data privacy and reidentification, any comparison against an aggregated summary view of the information exposure of all other participants would require that a minimum number of participants have completed the observation period. Only summary statistics aggregated across all participants are used to provide a baseline for the analytics, and no specific URLs or other identifiable information should be made available as part of the visualisation.

To avoid influencing participant behaviour during any study, visual analytics should only be made available after the observation period. Future research in the area might consider implementing visual analytics during a study as a form of intervention to modify levels of trust and for recommending new sources of information to study participants.

### Data linkage between information exposure and health behaviours via a web-based study platform

The web-based platform is designed to standardise the protocol for studies investigating associations between information exposure and health behaviours. In simple terms, the web-based platform manages the deployment of the active and passive data collection tools, supports the delivery of surveys, and stores and makes data available to study investigators for use in analysis. To be useful and taken up broadly by the community, it should be simple enough to encourage use by study investigators without specialised technical skills and in low resource settings.

Surveys are critical for linking information exposure of individuals to their health attitudes and behaviours. The web-based platform should make it easy for study investigators to deploy behavioural surveys (see Supplementary Information). While the questionnaires would be specific to the topic of the study, additional support might include lists of validated survey instruments that can be used for common topics such as vaccine hesitancy.

Data collection and storage should be centralised and hosted on a server to ensure security during data collection. Study investigators should also be required to register with the platform and agree to a set of terms and conditions to be able to develop and run a study. A critical condition would be ethics approval that includes a data privacy plan for any data that are transferred to the investigators. The centralisation of the web-based platform and data storage has advantages including clear processes for secure storage of highly sensitive data, and requirements for data privacy and ethics audits and checks throughout the life of the service. Centralised infrastructure can also support a scalable service that would allow for tens to thousands of study participants in individual studies, as well as the capacity to manage data for any number of studies and registered study investigators.

The web-based platform should also be flexible enough to support different kinds of studies and localised contexts. It should support different environments where information exposure may come from a broad range of online and offline sources. To do this, initial categories and subcategories should be easily defined by study investigators. Other areas requiring flexibility include supporting local requirements for ethics and recruitment, and patient information forms and informed consent processes. Using international standards as a basis, all studies should retain the ability to provide participants with the data that are collected from them and participants must retain their ability to revoke consent at any time during the study.

The expected process for a study would include a study investigator registering with the platform, confirming they have ethics approval, setting up a study by setting parameters, connecting to a questionnaire, and deciding on relevant keywords if using the passive data collection tool. Once the study has been created, the investigator uses generated study links as part of their external recruitment process. Recruitment and data collection continue over time and made available for investigators to download securely. Investigators would also be given an opportunity to prospectively opt in to sharing their data as part of large-scale meta-analyses examining information exposure and associations with health behaviours globally.

## Ethics and privacy considerations

Data acquired through both active and passive data collection and questionnaires is personal health data. As such, the platform should be able to handle data privacy and pass cybersecurity audits for all high-risk personal health data stored and made accessible to study investigators using the platform. The platform should also require that study investigators are registered to be able to conduct a study. A barrier to use of the platform should be set so that they must confirm that they agree to a set of requirements related to data privacy, ethics, and data retention. These would include requirements of local laws and expectations including ethics approvals and rights to data access and removal, and proof of local ethics approval should be cited as a condition of study registration.

As above, study investigators will be asked during the study design phase if they would like to contribute de-identified summary data to meta-analyses that measure the same health behaviour outcomes. If they agree, then de-identified metadata about timing, source, and trust will be recorded and made available for secondary use. Assuming the risk of re-identification is minimised in this process, access to aggregated and fully de-identified data from across studies could then be made available without any additional risks to data privacy.

Study investigators would be required to take responsibility for the privacy and security of data after they are downloaded as a package from the research platform. The platform would benefit from additional localised training about risks of reidentification and national and international laws related to participant rights of access to information, as well as any local requirements for ethics, data privacy, and data retention as needed. Where study investigators do not permit reuse of the summary data, any additional data that are not used as part of the summary data for meta-analyses (including photos, content, and URLs) can be permanently deleted from servers after a specified period and study investigators would then become responsible for any data retention requirements in their jurisdictions.

## Discussion

The rapid spread of health information, especially low-quality information, can have a negative impact on health outcomes by creating confusion and distrust, but it is challenging to measure associations between information exposure and health outcomes at scale and in robust ways. Tools to support measures of the burden of infodemics could be used to more directly inform where and when relevant public health interventions should be prioritised. We proposed and described connected tools that can be deployed as part of a web-based study platform that addresses this challenge.

The overall goal of the toolkit and web-based platform is to enable flexible but consistent study designs that investigate online and offline information exposure as factors associated with health attitude and behaviour outcomes. The expectation is that the platform will make the tools available for easy use by any research group, in ways that can be customised to match local context but constrained to match a well-defined protocol. This remains a challenge—to maintain a balance between flexibility to conduct a broad range of studies in localised contexts and ensuring that results data are reliable and synthesisable.

The specific design features of the toolkit and platform support its use across a global research collaboration, with a distributed approach to studies but with harmonised generation of data. This corresponds to a direct implementation of the WHO public health research agenda for managing infodemics, which includes generation of metrics for information exposure and health outcomes. The features of the toolkit that support this agenda include the standardised protocol implemented as part of the web-based platform, the open-source development and free access to the platform and its tools, and centralised storage of data which makes it simple for study investigators to prospectively opt-in to sharing de-identified data with the platform and community.

By standardising how information exposure is measured and supporting data sharing, the toolkit and platform can support globally coordinated prospective meta-analyses. We expect that this kind of meta-analysis will lead to more robust measures of information risk factors associated with health behaviours and global surveillance of the burden of infodemics. These measures will be a much more direct way to support policy and localised decision making about when and how to act.

There are limitations in the design of the platform and tools that could be addressed in future development and research efforts. The web-based platform currently requires internet access to be able to record and store exposures, but could be adapted to suit settings where internet access is limited. This would suit settings where offline information exposures make up the majority. There are limitations associated with sampling and selection biases in recruitment for prospective cohort studies. Participants willing to record and report on the information they access about certain topics may be a biased subset of populations of interest, and may under-sample from the most vulnerable populations. Other recruitment challenges might relate to people who have lower levels of digital literacy and do not feel comfortable using smartphone apps in their daily lives. A further limitation relates to the unknown effect of the tools on behaviour. While the tools are designed to support observational studies, the tools might affect information seeking, which may influence behaviour [22]. Evaluations of the tools in pilot studies should consider ways to measure or minimise the impact on information seeking behaviour.

## Conclusion

Infodemiology research faces major challenges because of the number of social media analysis studies focused on characterising online content on individual platforms without accounting for information exposure across platforms and offline, and linking data to health behaviours. To generate actionable evidence that can be used to guide the targeting and design of public health interventions, we need new ways to link observations of individual information exposure with health behaviours. We propose a solution that incorporates tools to measure information exposure through active or passive data collection, and a platform that helps researchers follow a standard protocol. By making the toolkit as broadly available as possible, we hope to shift the focus of infodemic research towards study designs that can be used in prospective meta-analyses that can directly support the global surveillance of infodemic burden.

## Supplementary Information

**Additional file 1:** **Supplementary Figure 1.** Study participant view of the media use diary tool on a smartphone screen. A standard approach for entering an example would be to add a new example, select an existingsource or a new source category, optionally describe or add links or images,rate the trust in the content, and submit. **Supplementary Figure 2.** Study investigator views of the web-based dashboard for developing and running a new study, with options to include the media use diary, online tracking tool, and links to surveys. A study investigator would name the study, include a brief description that participants will see, include a detailed participant information statement as required by local ethics requirements, and check the preview of how it would be displayed for participants. **Supplementary Figure 3.** Study investigator views of the web-based dashboard for developing and running a new study, with options to include the media use diary, online tracking tool, and links to surveys. For example, after selecting the category, an investigator can add or remove initial subcategories, and change the order of how they are initially displayed for participants. **Supplementary Figure 4.** Investigators can add links to surveys with questionnaires, designed to measure health behaviours using localised version of validated survey instruments. Investigators can choose when they want to ask participants to complete the survey, and link to the site for the survey which may vary between the start and end of the observation period. **Supplementary Figure 5.** Investigators control several parameters that determine which participants see browser popups asking them about trust. They first set the keywords, and then set up cohorts that represent a proportion of the participants and the rate at which they see the trust popup when they visit a site with relevant content identified by those keywords. **Supplementary Figure 6.**A complete study then has a URL that can be used in recruitment to provide participants with access to the toolkit and instructions on use.

## Data Availability

Data related to the development of the toolkit are available on request.
