# Supplementary material for: Measuring the burden of infodemics with a research toolkit for connecting information exposure, trust, and health behaviours
Source: Arch Public Health. 2023 Jun 5;81:102. doi: 10.1186/s13690-023-01101-7 (PMC10240452; doi:10.1186/s13690-023-01101-7)
Supplement: Supplementary file 1 — Additional file 1: Supplementary Figure 1. Study participant view of the media use diary tool on a smartphone screen. A standard approach for entering an example would be to add a new example, select an existingsource or a new source category, optionally describe or add links or images,rate the trust in the content, and submit. Supplementary Figure 2. Study investigator views of the web-based dashboard for developing and running a new study, with options to include the media use diary, online tracking tool, and links to surveys. A study investigator would name the study, include a brief description that participants will see, include a detailed participant information statement as required by local ethics requirements, and check the preview of how it would be displayed for participants. Supplementary Figure 3. Study investigator views of the web-based dashboard for developing and running a new study, with options to include the media use diary, online tracking tool, and links to surveys. For example, after selecting the category, an investigator can add or remove initial subcategories, and change the order of how they are initially displayed for participants. Supplementary Figure 4. Investigators can add links to surveys with questionnaires, designed to measure health behaviours using localised version of validated survey instruments. Investigators can choose when they want to ask participants to complete the survey, and link to the site for the survey which may vary between the start and end of the observation period. Supplementary Figure 5. Investigators control several parameters that determine which participants see browser popups asking them about trust. They first set the keywords, and then set up cohorts that represent a proportion of the participants and the rate at which they see the trust popup when they visit a site with relevant content identified by those keywords. Supplementary Figure 6.A complete study then has a URL that can be used in recruitment to [file 13690_2023_1101_MOESM1_ESM.docx]

**Supplementary Information**

The Information Diary Platform (IDP) is an open-source solution that includes a web-based platform for creating new studies that involve collecting data from participants via an active media use diary app, with or without a browser plugin for online tracking of relevant webpage access, and visual analytics to be used as an incentive at the end of an observation period. A fully functional demonstration system is currently available at http://idp-bidh.sydney.edu.au/ and the source code is available on GitHub.

***Participant view***

After a study has been created and participants are recruited into a study, they are given a unique code and provided access (and instructions) on how to participate in a study. Their unique code is linked to their email address, which is used as a unique identifier for linking external information to their information exposure data at the end of the study.

The simplest way for a participant to enter a new exposure is to click “New (+)”, select a category, select a subcategory, and then move the slider to indicate their level of trust in the information they see (***Supplementary Figure 1***). This includes a text description where they can also paste a URL from their browser or describe the information they saw in words, and an option to attach an image file or take a live picture. These are given as optional by default to avoid dropout and make it easy for participants to capture information exposures live.

The subcategories are adaptive and will change in order based on the frequency with which participants access each of the items in the subcategory. For example, if a participant most often engages with topic-specific information on Facebook, then Facebook will move to the top of the subcategory list.

Once the information has been entered by the participant, they can then submit the exposure and a confirmation screen is displayed to confirm that the submission was received and has been stored. In summary, once a participant has joined a study, they can record a topic-specific information exposure in as few as 5 clicks.


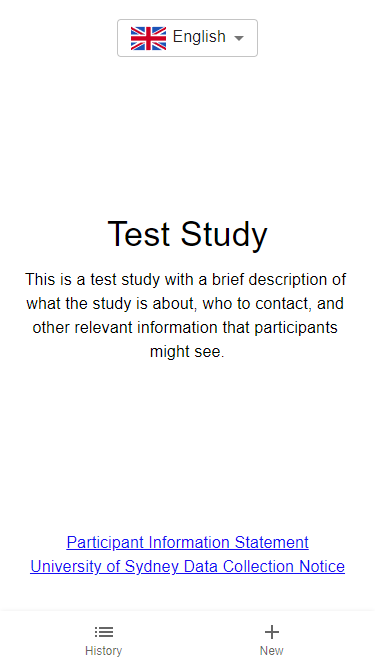

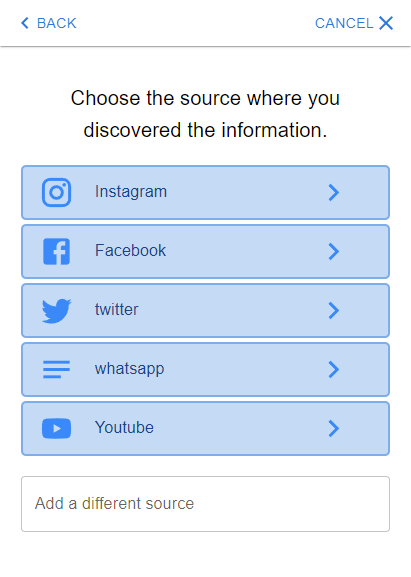

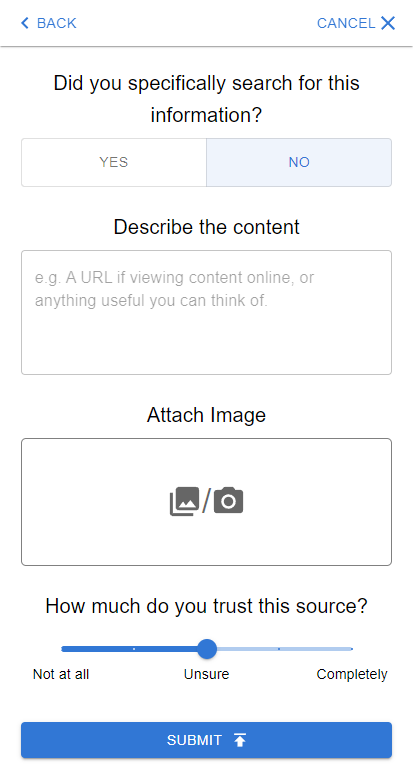


**Supplementary Figure 1.** Study participant view of the media use diary tool on a smartphone screen. A standard approach for entering an example would be to add a new example, select an existing source or a new source category, optionally describe or add links or images, rate the trust in the content, and submit.

***Study investigator view***

A registered study investigator can create multiple new studies and include basic information about the study title, description, and a detailed patient information statement (***Supplementary Figure 2***), as well as deciding whether to include either or both tools as part of the study. The patient information statement is appended with a statement from the platform explaining how the data will be securely stored, includes instructions on how to use the smartphone app on different smartphones and operating systems (the app is browser based and will work by connecting to a webpage and thus can be used on any device) and indicating that the responsibility for the ethics and data governance at the conclusion of the study belongs to the study investigator.


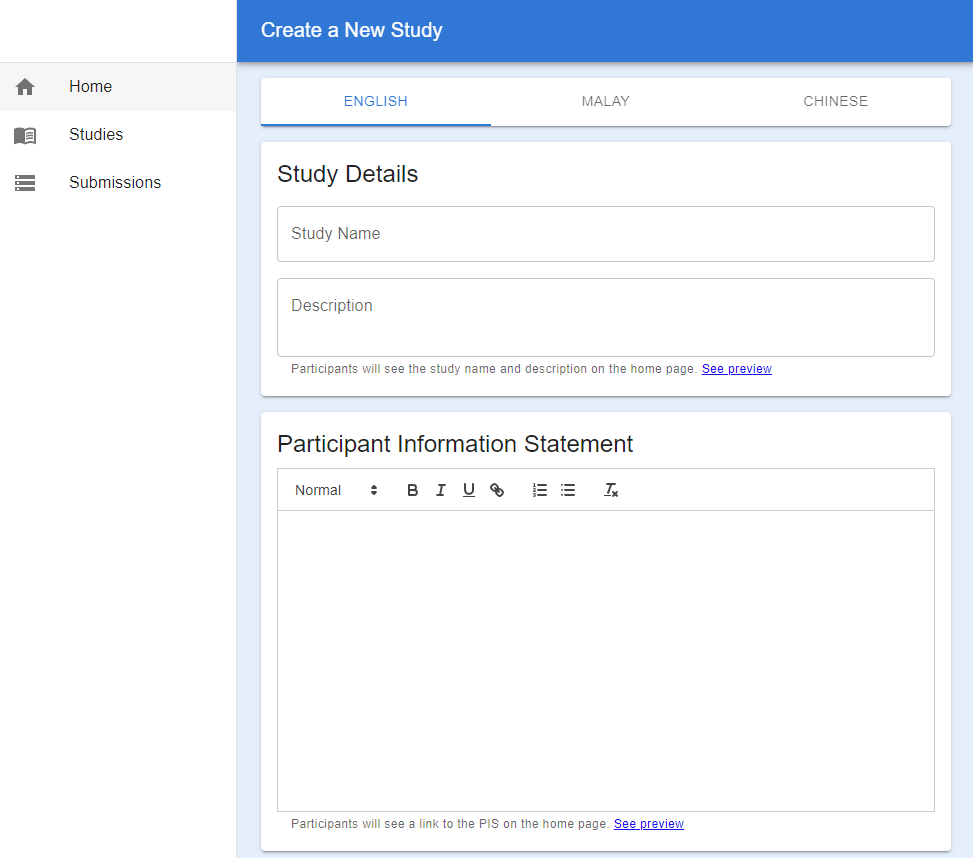


**Supplementary Figure 2.** Study investigator views of the web-based dashboard for developing and running a new study, with options to include the media use diary, online tracking tool, and links to surveys. A study investigator would name the study, include a brief description that participants will see, include a detailed participant information statement as required by local ethics requirements, and check the preview of how it would be displayed for participants.

For the media use diary, the study investigator can then set up and organise subcategories under the categories of “online search”, “online browsing”, and “offline” (traditional media and offline conversations). Common social media platforms and major platforms such as Facebook, YouTube, Instagram, Reddit, Twitter, WhatsApp, are all available with their icons, but study investigators can add any additional source and set an initial order (***Supplementary Figure 3***). Note that the order may change for study participants during use due to the inclusion of the adaptive reordering to reduce participant effort in recording examples.


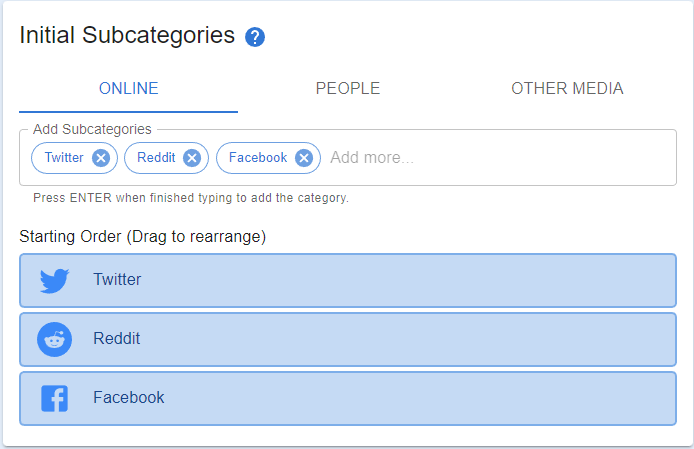


**Supplementary Figure 3.** Study investigator views of the web-based dashboard for developing and running a new study, with options to include the media use diary, online tracking tool, and links to surveys. For example, after selecting the category, an investigator can add or remove initial subcategories, and change the order of how they are initially displayed for participants.

The study investigator can also include links to external questionnaires (such as a Qualtrics survey) that will be sent to participants at specified times, generally at the start or end of the observation period (***Supplementary Figure 4***). Participants are connected via email accounts and no additional methods are used to avoid duplicates, so these standard study issues need to be managed by study investigators separately as part of their recruitment.


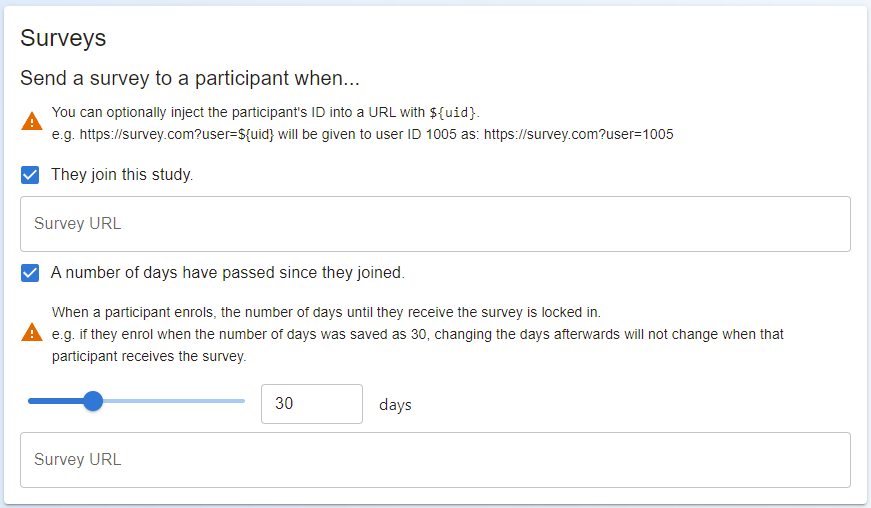


**Supplementary Figure 4.** Investigators can add links to surveys with questionnaires, designed to measure health behaviours using localised version of validated survey instruments. Investigators can choose when they want to ask participants to complete the survey, and link to the site for the survey which may vary between the start and end of the observation period.

For the online tracking, the study investigator enters a set of keywords that are used to identify relevant webpages for recording (***Supplementary Figure 5*)**. The investigator can then also organise study participants into cohorts by proportions and assign the popup frequency for each cohort differently. For example, half of the participants in the study may be asked about trust occasionally during the study (via a popup in their browser) while the other half of the participants in the study are never asked about trust for relevant webpages they visit.


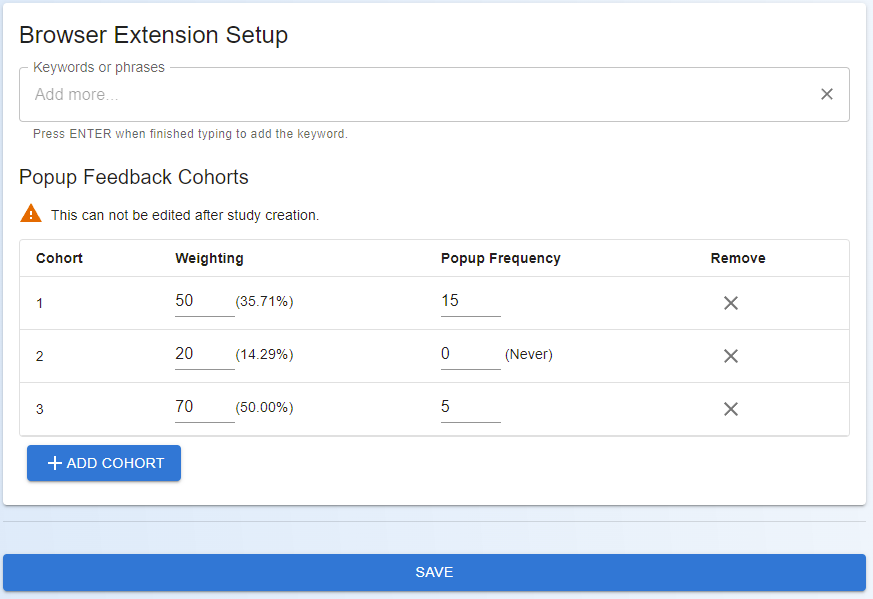


**Supplementary Figure 5.** Investigators control several parameters that determine which participants see browser popups asking them about trust. They first set the keywords, and then set up cohorts that represent a proportion of the participants and the rate at which they see the trust popup when they visit a site with relevant content identified by those keywords.

After a study has been created to include the passive online tracker including keywords and cohort construction, participants are provided with instructions for adding and activating a browser extension or plugin that is set up with a connection to a specific study (***Supplementary Figure 6***). Study participants can only participate in one study at a time and need to complete both steps for their browsers and devices.


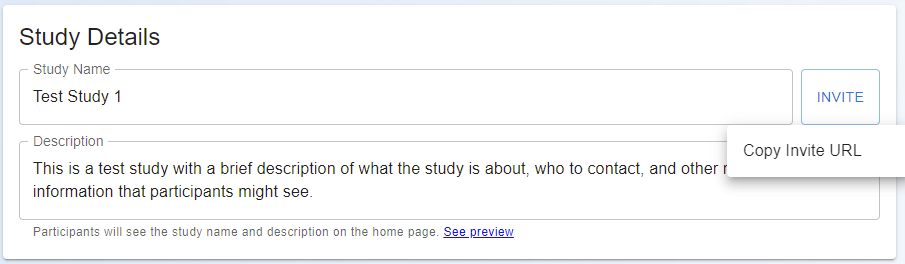


**Supplementary Figure 6.** A complete study then has a URL that can be used in recruitment to provide participants with access to the toolkit and instructions on use.
